# Docking analysis and the possibility of prediction efficacy for an anti-IL-13 biopharmaceutical treatment with tralokinumab and lebrikizumab for bronchial asthma

**DOI:** 10.1371/journal.pone.0188407

**Published:** 2017-11-20

**Authors:** Yutaka Nakamura, Aki Sugano, Mika Ohta, Yutaka Takaoka

**Affiliations:** 1 Department of Allergy and Rheumatology, Nippon Medical School Graduate School of Medicine, Sendagi, Tokyo, Japan; 2 Division of Medical informatics and Bioinformatics, Kobe University Graduate School of Medicine, Kobe, Japan; Ohio State University, UNITED STATES

## Abstract

Interleukin-13 (IL-13) is associated with allergic airway inflammation and airway remodeling. Our group found a variant with a single nucleotide polymorphism in the *IL13* gene at position +2044G>A (rs20541) that was expected to result in the non-conservative replacement of a positively charged arginine (R) with a neutral glutamine (Q) at position 144. IL-13Q144 was associated with augmented allergic airway inflammation and bronchial asthma remodeling. There is some indication that anti-IL-13 monoclonal antibodies can demonstrate a positive effect on the clinical course of refractory asthmatic patients. To date, the binding stability of these agents for IL-13Q144 is unknown. The objective of this study was to investigate the prediction efficacy of the anti-IL-13 monoclonal antibodies tralokinumab and lebrikizumab in asthmatic patients with IL-13R144 and IL-13Q144. The three-dimensional (3-D) structure of tralokinumab was obtained from the Protein Data Bank (PDB ID: 5L6Y), and the complete 3-D structure of lebrikizumab was built through homology modeling. For the binding stability analysis, we performed and analyzed docking simulations of IL-13 with tralokinumab or lebrikizumab. The tralokinumab and lebrikizumab structures changed after binding to IL-13 to facilitate binding with IL-13Q144. The stability analysis with tralokinumab and lebrikizumab demonstrated that IL-13Q144 was more stable than IL-13R144 for both the Rosetta energy score and for the free energy of binding. IL-13Q144 might be a promising predictor of responsiveness to tralokinumab and lebrikizumab treatment for bronchial asthma.

## Introduction

Bronchial asthma is a disorder of the conducting airways, which leads to variable airflow obstructions in association with airway hyper responsiveness and a local accumulation of inflammatory cells, particularly eosinophils, mast cells, and T lymphocytes [1]. Inhaled corticosteroids (ICSs) are the primary medication used to treat bronchial asthma, based on the efficacy of a strong anti-allergic agent on the inflammatory cells and induced mediators [2–3]. Although most asthmatic patients show a beneficial response to ICSs, there is great intra-individual variability in the treatment response level [4]. Th2-type cytokines, particularly interleukin-13 (IL-13), have been shown to orchestrate airway allergic inflammatory and remodeling processes [5–7], and anti-IL-13 agents have been highlighted for the treatment of asthma cases unresponsive to ICSs. Several clinical trials with biological agents against IL-13 have been conducted and have shown remarkable efficacy [8–9]. The clinical efficacy of anti-IL-13 antibodies may be influenced by the binding affinities of the antibodies to IL-13 and the molecular mechanisms through which they block IL-13 signaling. Polymorphisms of the *IL13* gene are associated with bronchial asthma [10], and atopy [11], as well as an elevated total serum IgE concentration [12–13]. Our group found a variant with a single nucleotide polymorphism (SNP) in the *IL13* gene at position +2044G>A (rs20541). This SNP is particularly interesting because it is found in approximately 25% of the general population and it is expected to result in the non-conservative replacement of a positively charged arginine (R) with a neutral glutamine (Q) at position 144 [10]. IL-13Q144 is associated with the airway remodeling of bronchial asthma [14], and it sequentially worsens patient quality of life. It should be noted that biopharmaceutical targeting IL-13 (lebrikizumab) has previously been shown to block IL-13 signaling via the IL-13 receptor (R) α1/ IL-4Rα. The binding stability and mechanism through which lebrikizumab blocks IL-13 signaling have been demonstrated [15], but the binding affinity of this agent for IL-13Q144 has not been described to date. Since we have previously confirmed that the correlation of the binding stability of gefitinib and the clinical data of lung cancer that contained an epidermal growth factor receptor mutation, the binding stability derived from structural analysis seems to aid the prediction of the drug efficacy [16]. In this study, we analyzed the IL-13 variant based on the binding stability of the anti-IL-13 antibodies tralokinumab and lebrikizumab.

## Methods

### Three-dimensional structures of tralokinumab and lebrikizumab

**Fig 1:**
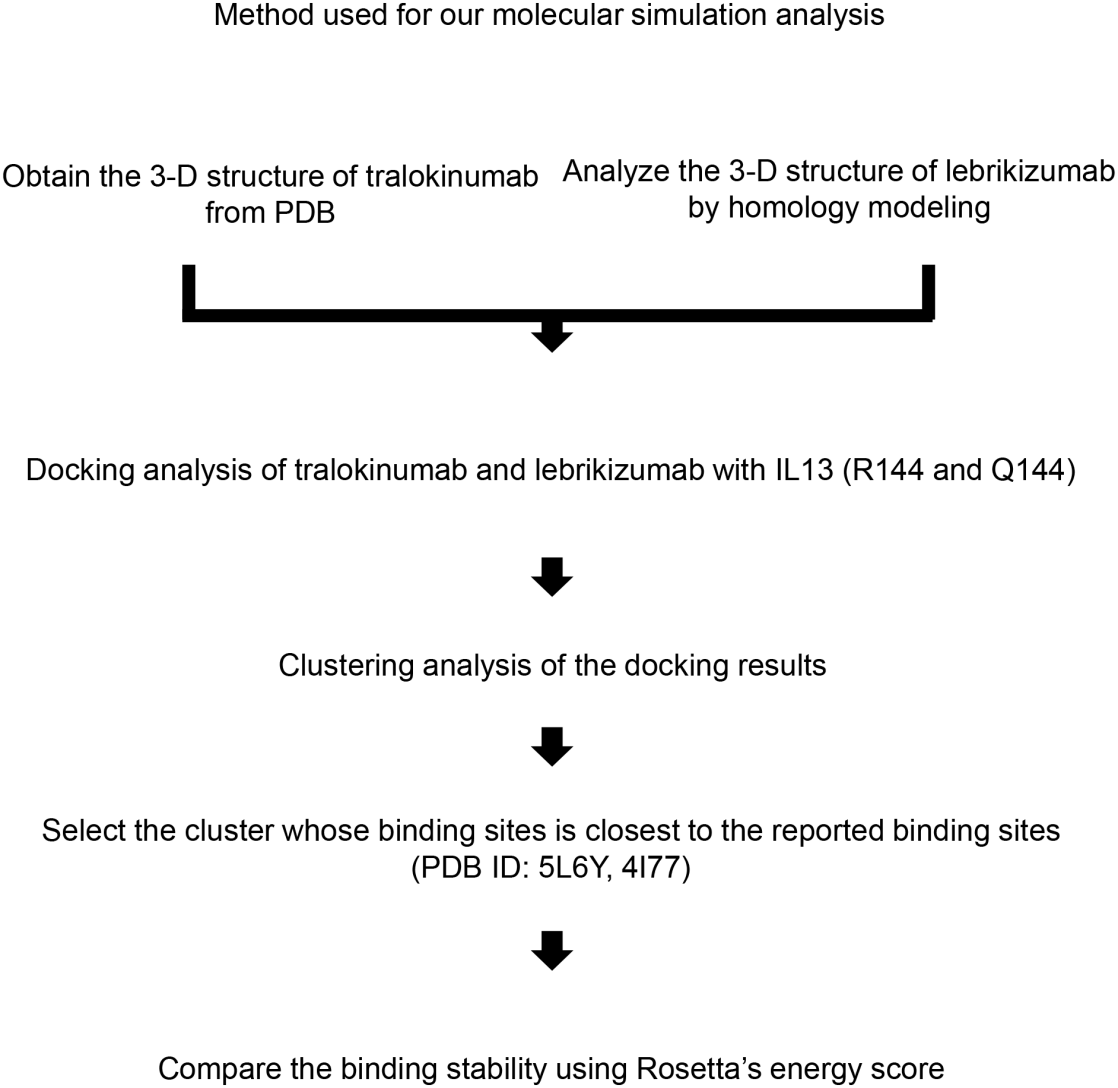
The method used for the molecular simulation analysis. 3-D, three-dimensional; PDB, Protein Data Bank; R144, arginine (R) at position 144 of IL-13 protein; Q144, replacement of a positively charged arginine (R) with a neutral glutamine (Q) at position 144 of IL-13 protein.

The 3-D structure of tralokinumab was obtained from the Protein Data Bank (PDB ID: 5L6Y). The complete 3-D structure of lebrikizumab was generated using homology modeling because the Fc domain was not included in the crystal structure available in the PDB (ID: 4I77) (Fig 1). For the homology modeling, the amino acid sequence of lebrikizumab was obtained from the ChEMBL database (Compound ID: CHEMBL1743035). Using the heavy chain sequence, we performed a BLAST search against the PDB. Based on the sequence identity (83% identity), PDB ID 5DK3 was selected as the template structure for the lebrikizumab heavy chain, including the Fc domain. The 3-D structure of the heavy chain was generated using the homology modeling function in the MOE software (Chemical Computing Group, Quebec, Canada) and was superimposed on the Fab domain of lebrikizumab (PDB ID: 4I77). The resulting heterodimeric structure was subjected to structural optimization using GROMACS software [17] with the AMBER99 force field. The TIP3P water model [18] was used to solvate the protein and counter-ions. The minimum distance of a protein atom to the edge of the rectangular water box was 14Å. Na^+^ and Cl^−^ ions were added to keep the whole system neutral, leading to salt concentrations of 0.15 M. Energy minimization was carried out using the steepest descent method until the maximum force on any atom (Fmax) was less than 1000 kJ/mol/Å. The systems were then heated to 310 K during 250 ps. After the heating process, a 5,000 ps production run was performed with the NPT ensemble, [i.e., a constant number of particles (N, 453,975 atoms), pressure (P, 1 atm), and temperature (T, 310 K)] in a unit of 2 fs and particle mesh Ewald (PME) option. The temperature and pressure of the system were maintained using the Berendsen coupling algorithm [19]. All bond lengths involving H atoms were constrained with the LINCS algorithm [20]. The root-mean-square deviation (RMSD) from the 5000 ps molecular dynamics trajectory were analyzed by using the rmsdist GROMACS inbuilt tool. The molecule in the last frame was used for the subsequent analysis. The quality of the lebrikizumab structure was ascertained using the PROCHECK program [21]. The PROCHECK yielded 87.0, 10.9, 2.3, and 0.7% in the most favored, allowed, generously allowed, and disallowed regions of the Ramachandran plot, respectively. This provided an insight into the correctness of the modeled structures in terms of PROCHECK (S1 Fig).

### Docking analysis of IL-13 with tralokinumab and lebrikizumab

For the binding stability analysis, we performed and analyzed the docking simulation of IL-13 with tralokinumab and lebrikizumab. The 3-D structures of human IL-13 were downloaded from the PDB. We chose PDB ID: 4PS4, which contains the largest number of residues among the 3-D structures of human IL-13R144 in the PDB, for IL-13R144. We also chose PDB ID: 3BPO for IL-13Q144 because only this structure had a 3-D structure of human IL-13Q144 amongst the PDB entries containing IL-13 with residue 144. The chain and residues of the docking sites are as follows: H:Ser103, H:Trp104, L:Ser1, L:Gly24, L:Asn25, L:Ile26, L:Gly28, L:Ser29, L:Lys30, L:Leu31, L:Ser66, L:Gly67 and L:Gly93 for tralokinumab; and H:Ser28, H:Ser30, H:Ala31, H:Tyr32, H:Gly53, H:Gly99, H:Tyr100, H:Tyr101, H:Pro102 and L:Ser31 for lebrikizumab. In total, 100 flexible docking runs were performed using Rosetta software with side-chain optimization and energy minimization [22]. The most stable complex was selected for each IL-13—biopharmaceutical pair. The RMS deviation between the most stable docking result of each IL-13—biopharmaceutical pair and the corresponding crystal structure were as follows: tralokinumab with IL-13R144, 1.66 Å; tralokinumab with IL-13Q144, 2.20 Å; lebrikizumab with IL-13R144, 8.25 Å; and lebrikizumab with IL-13Q144, 4.90 Å. These docking results, which were similar to the crystal structure poses, were applied to the subsequent analysis. These IL-13-biopharmaceutical complexes were subjected to 5000-ps MD simulations as described in the previous section, and the binding free energies were analyzed by using g_mmpbsa software [23].

## Results

### Molecular dynamics simulation of lebrikizumab

The 3-D structure of lebrikizumab from homology modeling was optimized using an MD simulation in solvent to mimic the real physiological environment. The stability of the lebrikizumab structure during the MD simulation was measured by its deviation from the initial structure in terms of RMSD. The structural optimization of this MD simulation succeeded because the 3D structure of lebrikizumab reached a stable state after 3500 ps where the RMSD converged to 2.9 Å (S2 Fig).

### Docking simulation of tralokinumab and lebrikizumab with IL-13

**Fig 2:**
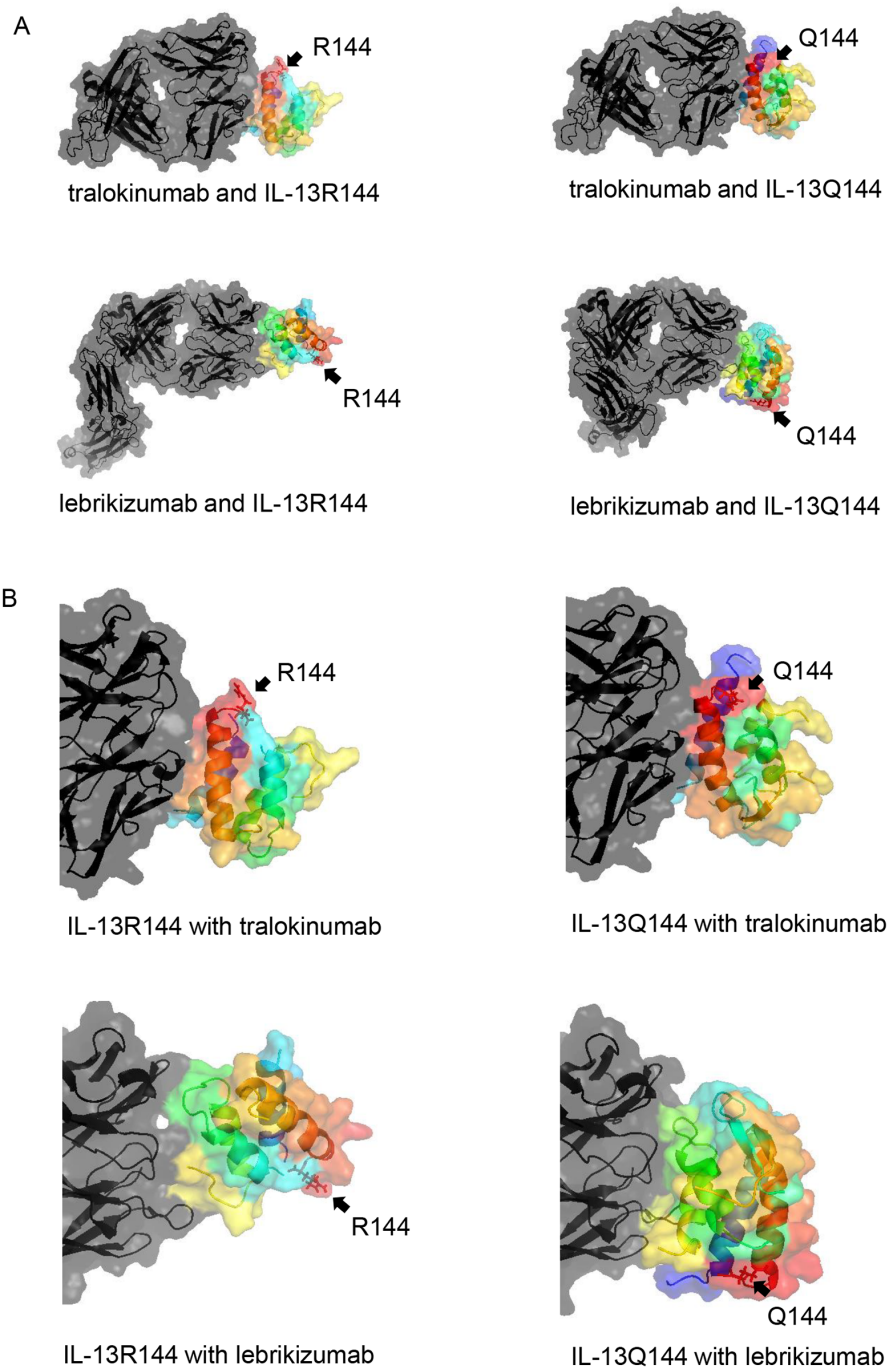
The three-dimensional binding structures of tralokinumab, lebrikizumab, and IL-13. (A) A whole structure view and (B) a close-up view of IL-13 are shown via surface and cartoon representations. The tralokinumab and lebrikizumab structures were transformed after they bound with IL-13. The gradation colour of IL-13 was displayed using the spectrum function of PyMOL software, thus colouring the N-terminal to C-terminal domains, in order, as blue, cyan, green, yellow, orange, and red. Tralokinumab and lebrikizumab, including complementarity determining regions, were depicted as black. Residues at position 144 of IL-13 protein were indicated by arrows and shown in stick representation.

We identified the 3-D structures of tralokinumab, lebrikizumab, IL-13R144, and IL-13Q144. The tralokinumab and lebrikizumab structures were transformed following their binding with IL-13 (Fig 2). Note that the binding sites of these biopharmaceuticals with IL-13 differed.

### Binding stability of tralokinumab and lebrikizumab with IL-13

Next, we analyzed the binding stability of the biopharmaceuticals with IL-13R144 and IL-13Q144. The stabilities of tralokinumab and lebrikizumab with IL-13R144 or IL-13Q144 differed. Moreover, the binding was more stable with IL-13Q144 than with IL-13R144 for both the Rosetta energy score and the free energy of binding (Table 1).

**Table 1:** Binding stability of tralokinumab and lebrikizumab with IL-13.

	Rosetta’s energy score (REU)		Total energy (kcal/mol)	
	complex with IL-13R144	complex with IL-13Q144	complex with IL-13R144	complex with IL-13Q144
tralokinumab	-1522.531	-1549.034	-219.950	-276.692
lebrikizumab	-2122.571	-2158.778	-110.031	-178.468

IL-13R144, wild type of IL-13, which contains arginine (R) at position 144 of the IL-13 protein. IL-13Q144, executes a non-conservative replacement of a positively charged arginine (R) with a neutral glutamine (Q) at position 144 of the IL-13 protein.

## Discussion

In this study, we performed a molecular simulation analysis of the biopharmaceutical anti-IL-13 antibodies tralokinumab and lebrikizumab. Several new findings emerged from this study. First, the 3-D models differentiated IL-13R144 from IL-13Q144 and showed an alteration of the protein molecular conformation of tralokinumab and lebrikizumab after binding with IL-13R144 and IL-13Q144. Second, we demonstrated that the binding of tralokinumab and lebrikizumab with IL-13Q144 was more stable than the binding with IL-13R144.

Tralokinumab, a human IL-13 neutralizing monoclonal IgG4 antibody, has been tested in patients with uncontrolled asthma [24], ulcerative colitis, [25] and rapidly progressive idiopathic pulmonary fibrosis [26]. Tralokinumab blocks the signaling pathway through the IL-4Rα/IL-13Rα1 heterodimer by blocking soluble IL-13, thereby preventing a link to the receptor. In an early study of moderate-to-severe asthma, 194 patients (92% of whom were white) were randomized at 27 sites in Europe. The addition of tralokinumab showed no significant improvement in the Asthma Control Questionnaire’s mean of six individual item scores; however, an effect of tralokinumab on force expiratory flow volume in one second (FEV1) was observed [27]. Another phase 2b study of tralokinumab showed a nonsignificant reduction of the asthma exacerbation rates, and somewhat encouraging results were observed in a subgroup of patients with higher baseline levels of dipeptidyl peptidase (DPP)-4 and periostin [8]. DPP-4 has been demonstrated to be derived from bronchial epithelial cells in the presence of IL-13 [27]. Additionally, periostin is an IL-13-induced matricellular protein that is basally secreted from bronchial epithelial cells; it can be detected in serum but not in bronchial lavage fluid [28]. Lebrikizumab is an IgG4 humanized monoclonal antibody that also prevents soluble IL-13 from binding with IL-4Rα/IL-13Rα1. Because asthmatic patients with high serum periostin concentrations exhibit improved lung functions and reduced exacerbation with lebrikizumab, the serum periostin level has been considered to be a predictor of the response to anti-IL-13 therapy [9, 29]. Asthmatic patients with high serum periostin concentrations have been shown to benefit the most from anti-IL-13 treatment in the 2 clinical studies with lebrikizumab. Nevertheless, another study did not demonstrate the efficacy of lebrikizumab on increased FEV1 in patients with high periostin levels [30]. These data support the heterogeneous nature of asthma and the need for discrimination, even in the presence of a high periostin concentration as a predictive biomarker of the response to lebrikizumab. The clinical trials of the LAVOLTA studies, which were designed to assess the rate of severe asthma exacerbations over 52 weeks in a group with high serum periostin concentrations, enrolled and treated 2148 patients from 28 countries in the northern and southern hemispheres [31]. Two trials (LAVOLTA I and LAVOLTA II) were simultaneously conducted; however, lebrikizumab was shown to reduce exacerbation rates in periostin-high patients in LAVOLTA I but not in LAVOLTA II. Several factors may explain this discrepancy. Foremost, the patients enrolled in the two trials included various ethnic populations, with different genetic backgrounds. The A allele frequency in the *IL13* gene at position +2044 G has been shown to have a relatively wide range from 15.3% to 62.9% across different ethnic populations [10, 32]. For instance, it has also been shown that the genetic variance rate of US residents with Mexican ancestry extends to 51.2%, however, the genetic variance among African Americans is much lower at 18.9%. This variance rate does not depend on the country in which they are residing but rather upon ancestry. When *wt IL13* was identified in a relatively large proportion of the people who participated in the clinical trial, a reduced binding affinity was noted for lebrikizumab; therefore, because the trial did not produce the expected results, further examinations are needed to verify the relationship between *wt* and mutant *IL13* and the clinical data of the enrolled asthmatic patients. Hopefully, the genetic back-grounds of the enrolled patients will become more apparent. Previous *in vitro* studies have demonstrated that IL-13Rα2 has a lower affinity for IL-13Q144 and, thus, is unable to regulate IL-13 as effectively as IL-13R144 [33]. Because this lower affinity leads to a more sustained response than that observed for IL-13R144 [14, 34], the induction of the periostin and DPP-4 levels by IL-13 could be increased for asthmatic patients with IL-13Q144. Thus, the governing and controling responsiveness to anti-IL-13 antibodies may depend on IL-13 characteristics rather than IL-13-induced molecules. A potential limitation of this study should be noted. We did not analyze the binding kinetics of IL-13R144 and IL-13Q144 to lebrikizumab and tralokinumab *in vitro*. However, we assume that structural and binding stability are also important as part of the approach to better predict the effects of molecule-targeting therapeutic agents [16] and biopharmaceuticals.

In conclusion, when tralokinumab and lebrikizumab bound IL-13, their structures changed to facilitate their binding stability with IL-13Q144. Therefore, IL-13Q144 might be a promising and complementary genomic biomarker as a predictor of tralokinumab and lebrikizumab responses to bronchial asthma.

## Supporting information

S1 Fig PROCHECK Ramachandran scores for the structures of lebrikizumab. (PPTX)

S2 Fig RMSD value relative to the initial structure as a function of simulation time. (PPTX)
