# Supplementary material for: Docking analysis and the possibility of prediction efficacy for an anti-IL-13 biopharmaceutical treatment with tralokinumab and lebrikizumab for bronchial asthma
Source: PLoS One. 2017 Nov 20;12(11):e0188407. doi: 10.1371/journal.pone.0188407 (PMC5695818; doi:10.1371/journal.pone.0188407)
Supplement: S1 Fig — (PPTX) [file pone.0188407.s001.pptx]

## Slide 1
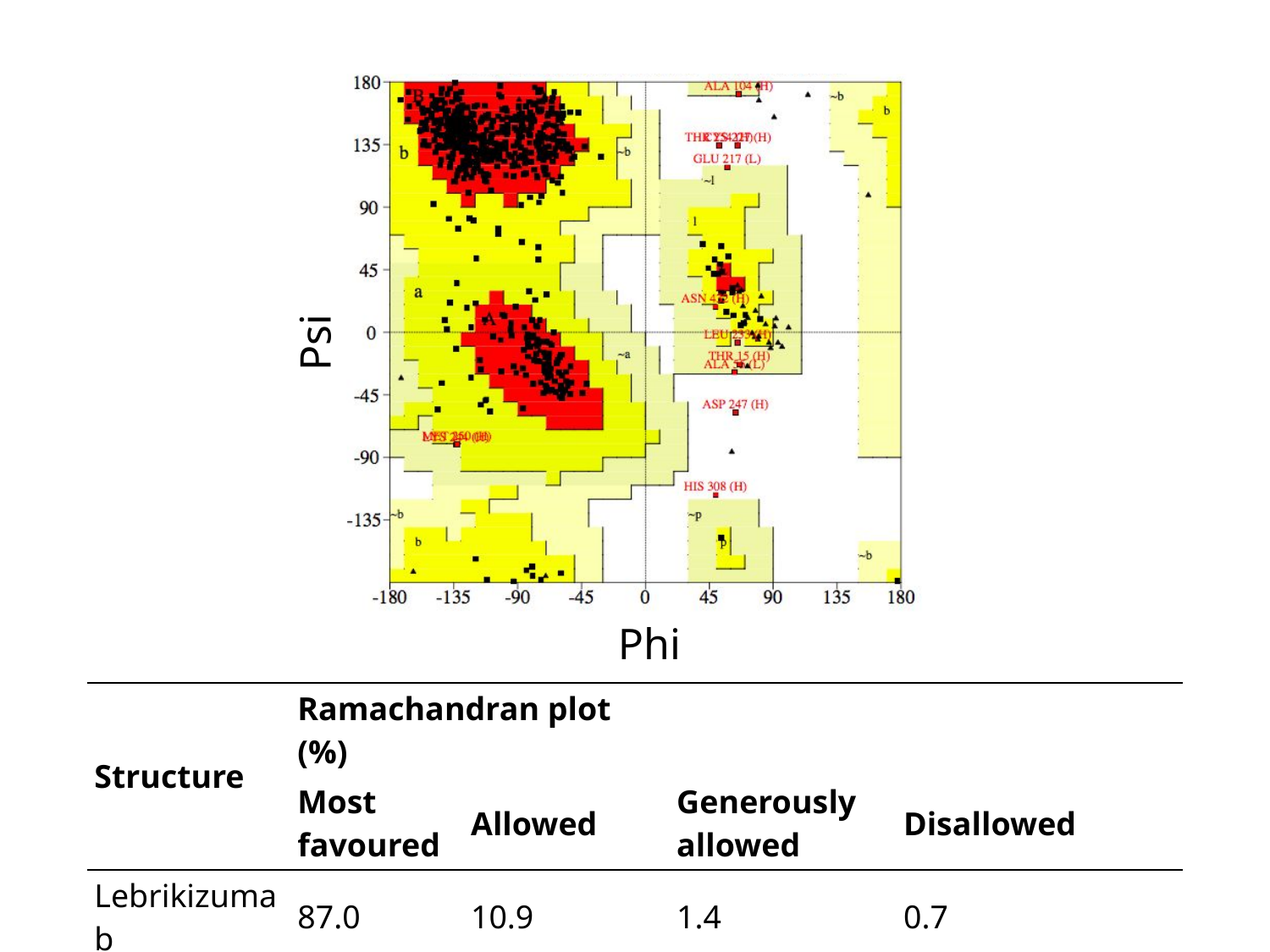

Psi
Phi
| Structure | Ramachandran plot (%) | | | |
| --- | --- | --- | --- | --- |
| | Most favoured | Allowed | Generously allowed | Disallowed |
| Lebrikizumab | 87.0 | 10.9 | 1.4 | 0.7 |
