# Supplementary figures and images for: Docking analysis and the possibility of prediction efficacy for an anti-IL-13 biopharmaceutical treatment with tralokinumab and lebrikizumab for bronchial asthma
Source: PLoS One. 2017 Nov 20;12(11):e0188407. doi: 10.1371/journal.pone.0188407 (PMC5695818; doi:10.1371/journal.pone.0188407)

## Slide 1
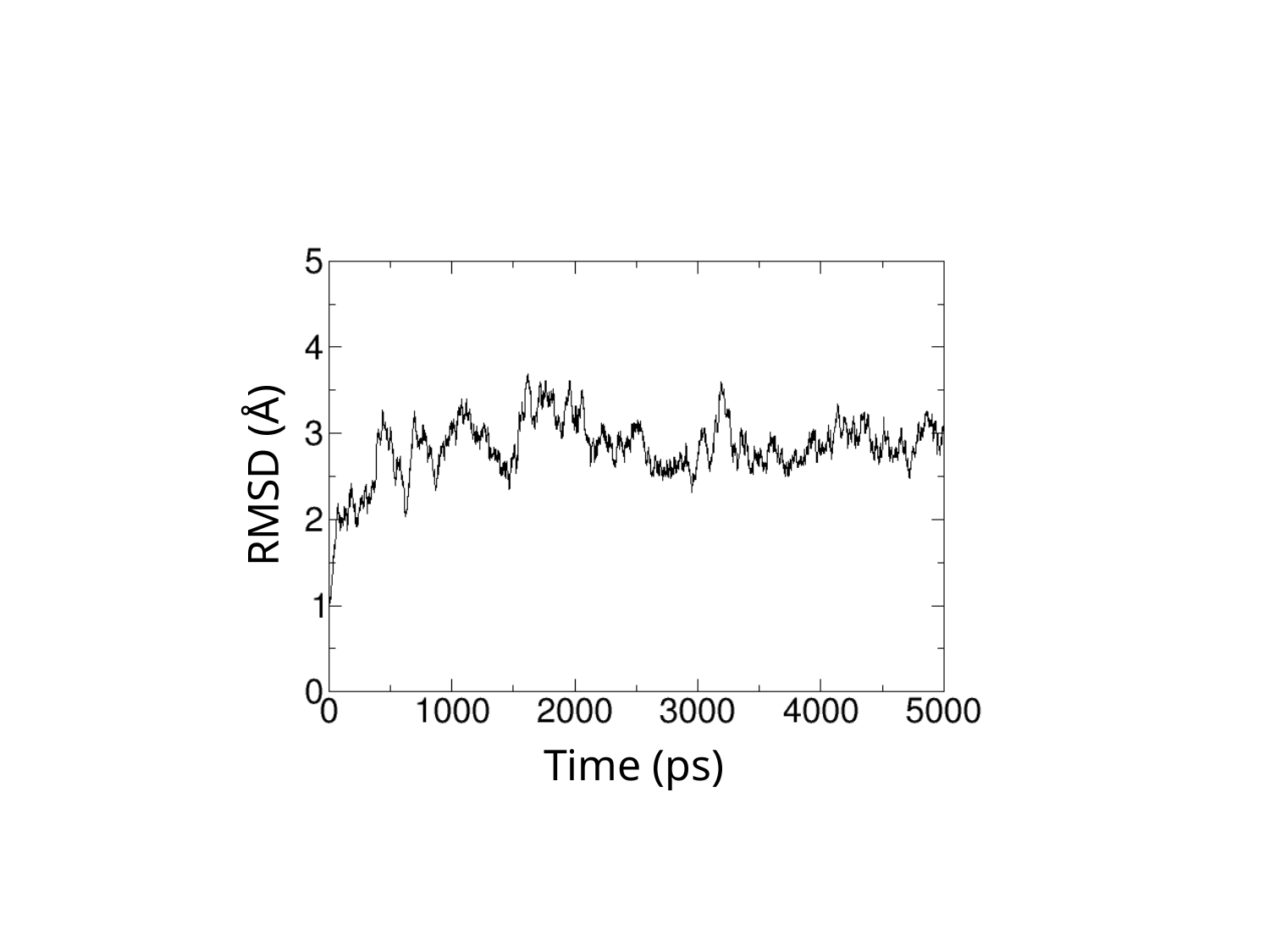

RMSD (Å)
Time (ps)

Supplement: S2 Fig — (PPTX) [file pone.0188407.s002.pptx]
